# Supplementary material for: Developmental trajectory and evolutionary origin of thymic mimetic cells
Source: Nature. 2025 Jun 11;643(8073):1066–75. doi: 10.1038/s41586-025-09148-y (PMC12286861; doi:10.1038/s41586-025-09148-y)
Supplement: Supplementary file 1 — This file contains Supplementary Tables 1–4 and Supplementary Figs. 1–8. [file 41586_2025_9148_MOESM1_ESM.pdf]

nature portfolio

https://doi.org/10.1038/s41586-025-09148-y

Supplementary information

Developmental trajectory and evolutionary origin of thymic mimetic cells

In the format provided by the authors and unedited

Supplementary Information

for

Developmental trajectory and evolutionary origin of thymic mimetic cells

Anja Nusser, Oliver S. Thomas, Gaoqun Zhang, Daisuke Nagakubo, Laura Arrigoni, Brigitte Krauth & Thomas Boehm

Supplementary Table 1 | Enumeration of TEC subpopulations at various developmental time points.

Population	E16.5	P0	P28
Early Progenitor	719 (32.9%)	226 (13.0%)	296 (4.3%)
Postnatal Progenitor	128 (5.9%)	113 (6.5%)	2242 (32.2%)
cTEC	1156 (52.9%)	921 (52.8%)	445 (6.4%)
cTEC (nurse)	0 (0.0%)	70 (4.0%)	277 (4.0%)
mTEC	0 (0.0%)	37 (2.1%)	1044 (15.0%)
unassigned	139 (6.4%)	75 (4.3%)	453 (6.5%)
Aire-stage	42 (1.9%)	268 (15.4%)	1528 (22.0%)
Ciliated	0 (0.0%)	3 (0.2%)	49 (0.7%)
Goblet	2 (0.1%)	4 (0.2%)	31 (0.4%)
Ionocyte	0 (0.0%)	0 (0.0%)	13 (0.2%)
Muscle	1 (0.0%)	9 (0.5%)	10 (0.1%)
Enterohepatic	0 (0.0%)	1 (0.1%)	82 (1.2%)
Lung (basal)	0 (0.0%)	0 (0.0%)	31 (0.4%)
Microfold	0 (0.0%)	1 (0.1%)	57 (0.8%)
Neuroendocrine	0 (0.0%)	2 (0.1%)	32 (0.5%)
Pancreatic	0 (0.0%)	1 (0.1%)	52 (0.7%)
Skin	0 (0.0%)	7 (0.4%)	86 (1.2%)
Tuft	0 (0.0%)	7 (0.4%)	231 (3.3%)

Supplementary Table 2 | Genotyping information

Transgene	Forward primer	Reverse primer	Size (bp)
Foxn1 (wt)	5'-CTGTGAACTCAGCCATACTC	5'-TGCACCAAGCCTCTGCTGGGA	521
Foxn1 (mut)	5'-TCGCCTTCTTGACGAGTTCT	5'-TGCACCAAGCCTCTGCTGGGA	230
Foxn1:Cre	5'-TGCATGATCTCCGGTATTGA	5'-CGTACTGACGGTGGGAGAAT	374
Rosa26LSLEYFP (wt)	5'-AAAGTCGCTCTGAGTTGTTAT	5'-GGAGCGGGAGAAATGGATATG	600
Rosa26LSLEYFP (del)	5'-AAAGTCGCTCTGAGTTGTTAT	5'-GCGAAGAGTTTGTCCTCAACC	300
Foxn1:mCardinal	5'-GTCCCTAATCCGATGGCTAGCTC	5'-GTAGATCAAGCAGCCGTCCT	492
Foxn1:Bl_Foxn4	5'-CCAGCTCCGAAACAGCCTAA	5'-GTCCTTTGTCGTCTGGTCGT	531
Foxn1:Cm_Foxn4	5'-GTCCCTAATCCGATGGCTAGCTC	5'-GGTTAAAGTTCATGCGGCCG	374
Foxn1:Cm_Foxn1	5'-GTCCCTAATCCGATGGCTAGCTC	5'-TATCGCGTGCACGAGTTGTA	397
Foxn1:Fgf7	5'-CCGTGGCAGTTGGAATTGTGGC	5'-ACATTTCCCCTCCGCTGTGTGTC	186
Foxn1:Bmp4	5'-CATTGTAGCTGGCTTTCTTCGAG	5'-GATCTCAGCGGCATCCAC	1523
Foxn1:Δ3ex2_Foxn1	5'-GCATGCTAACTTCAGCTGC	5'-CACTGTCCAGAGCTTG	278
Ascl1 (wt)	5'-CTACTGTCCAAACGCAAAGTGG	5'-GCTCCCACAATCCTCGTAAAGA	400
Ascl1 (mut)	5'-CTACTGTCCAAACGCAAAGTGG	5'-TAGACGTTGTGGCTGTTGTAGT	1000

Supplementary Table 3 | Primers for RT-PCR amplifications for *C. punctatum*

Gene	Forward Primer	Reverse Primer	Size (bp)
Pax9	5′-CAAACCAGGCTGCAGTGAATGG	5′-GACACAAAGCTGCTAGCTGTAGG	102
Foxn1	5′-CAACACAGATATTGATGCACTC	5′-CCATGGAGAAGACATGGACAC	123
Foxn4	5′-GACGTTGATGCCCTTGACCCAACC	5′-GCTAAATGCACTGAGTGTGTCC	108
Trac	5′-CACCATCTGAACCATCCTTGTACG	5′-CTCATGGTCATGTTATAGCTCTTG	135
Trbc	5′-CCTCACCAGATGAGATTAAACAG	5′-TTTTGATATTGTCAGGATAGAAG	81
IgK	5′-ACTGTGAAGTATGTGGAAATATCAG	5′-CAAAAGTTTTGTCTTCCAAAGGTGAG	86
Dll4	5′-GATCTGACTCAAGACCTGAAATG	5′-AACTGTGGATATTCTGCACTCTGG	105
Foxa1	5′-AGGACATGAAGCTTCTGACTGG	5′-CTGAAGGAGCCCGAGTTCAT	162
Pou2f3	5′-CATCATCACCAGTTACCACATCC	5′-GTGCAAGAACGAGGTACCATTCC	116
Trpm5	5′-GAAGAAGACATCAACCAAGGTAG	5′-CATCCAGGATAGAACATCACTGC	116
Aire	5′-CTCCGCTAAAACGGAAATCTCC	5′-CTGTTATCTTTTTGAACTGGTTGTG	187
MyoG	5′-TCCTCTGACCATTGCAACACTTCG	5′-TGAGAGAACACAAGCTGGTACTTCC	99
Foxj1b	5´-CAACTTCTGCTACTACAAGCACGCTG	5′-ACCAGGTTCATCTTTCTGTCTTGGCAC	114

Supplementary Table 4 | Primers for RT-PCR amplifications for *D. rerio*

Gene	Forward Primer	Reverse Primer	Size (bp)
pax9	5'-TCCTCGGGAGGCTAAACGCAATGG	5'- GCTGATGCACAAGGCCTGATGC	149
foxn1	5'-TACATTAGGCACTGATGTGGAGACC	5'-ACTGATGGCACTGTATGCCTGATGG	130
foxn4	5'-GTCCACACTGACGTCAATTCAGAGG	5'-GCTGCTGGTGTCCAGGTCACAGTCG	174
psmb11	5'-GCCAAACTCCTGTCCCACATGCTGC	5'-GCATATCTCTCTGTCATTGGTTGCTCG	125
aire	5'-CAGAGACGAGATGGACTCAGTGATGG	5'-CTTGGGTCTCAGAAAGAGGACGTG	98
ef1a	5'-TATCTCCAAGAACGGACATAC	5'-GCAAACTTGCAGGCGATGTG	740

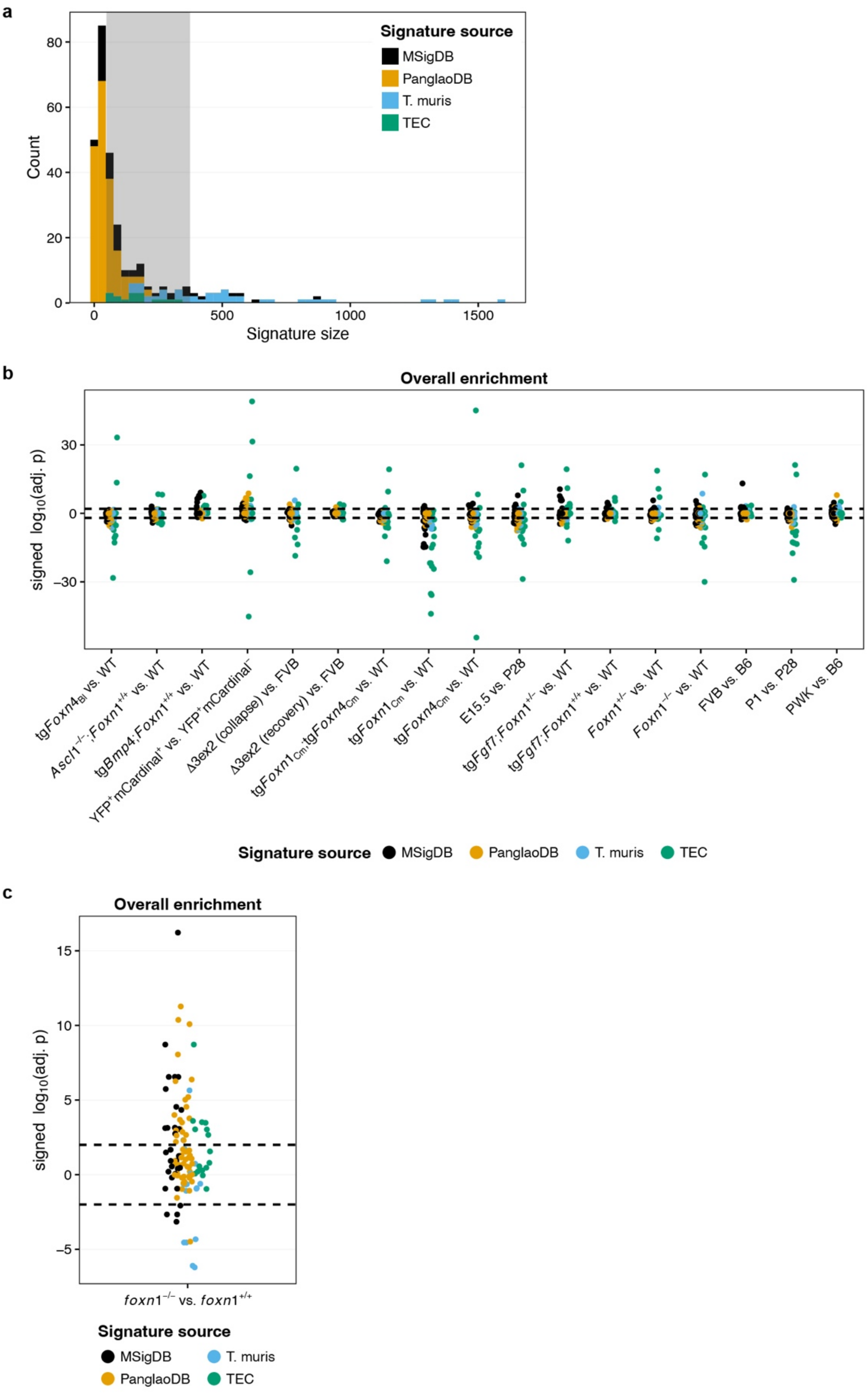

Supplementary Figure 1 | Markers for enrichment analysis. a, In addition to signatures from canonical TEC and mimetic populations, background cell type signatures were collected from different databases as indicated. The signature size is the number of genes comprising the signature. Only signatures in the size range of the TEC signatures (± 10%, within the shaded area) were retained. **b**, Overall enrichment in all mouse bulk RNAseq data discussed in this study. Dashed lines indicate p = 0.01. Signatures above the upper line are upregulated,

those below the lower line are downregulated. TEC signatures reached significant *p* values more frequently than non-TEC signatures. **c**, Equivalent plot showing overall enrichment in zebrafish bulk RNAseq samples.

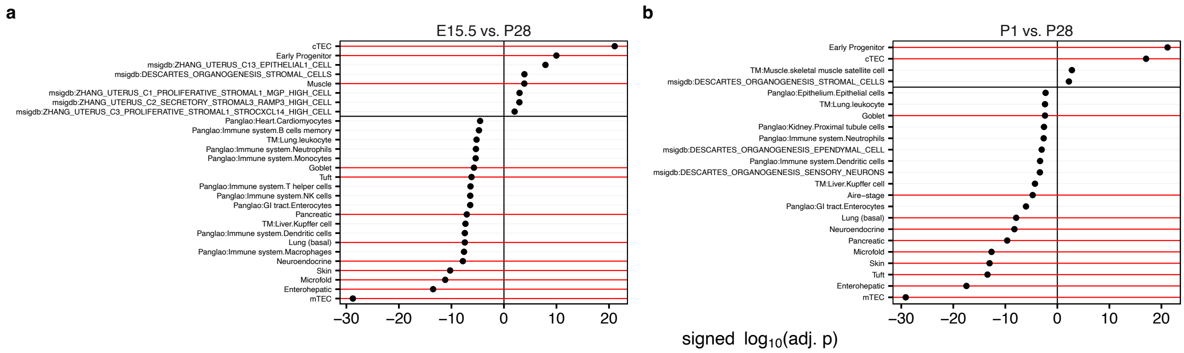

Supplementary Figure 2 | Top enriched signatures for E15.5 and P1 vs. P28. Plots show signed and logarithmized *p* values of up to 20 up- and up to 20 downregulated signatures. Only signatures with enrichment $p < 0.01$ are shown. Negative values indicate downregulation, positive values indicate upregulation. Red lines mark TEC signatures,

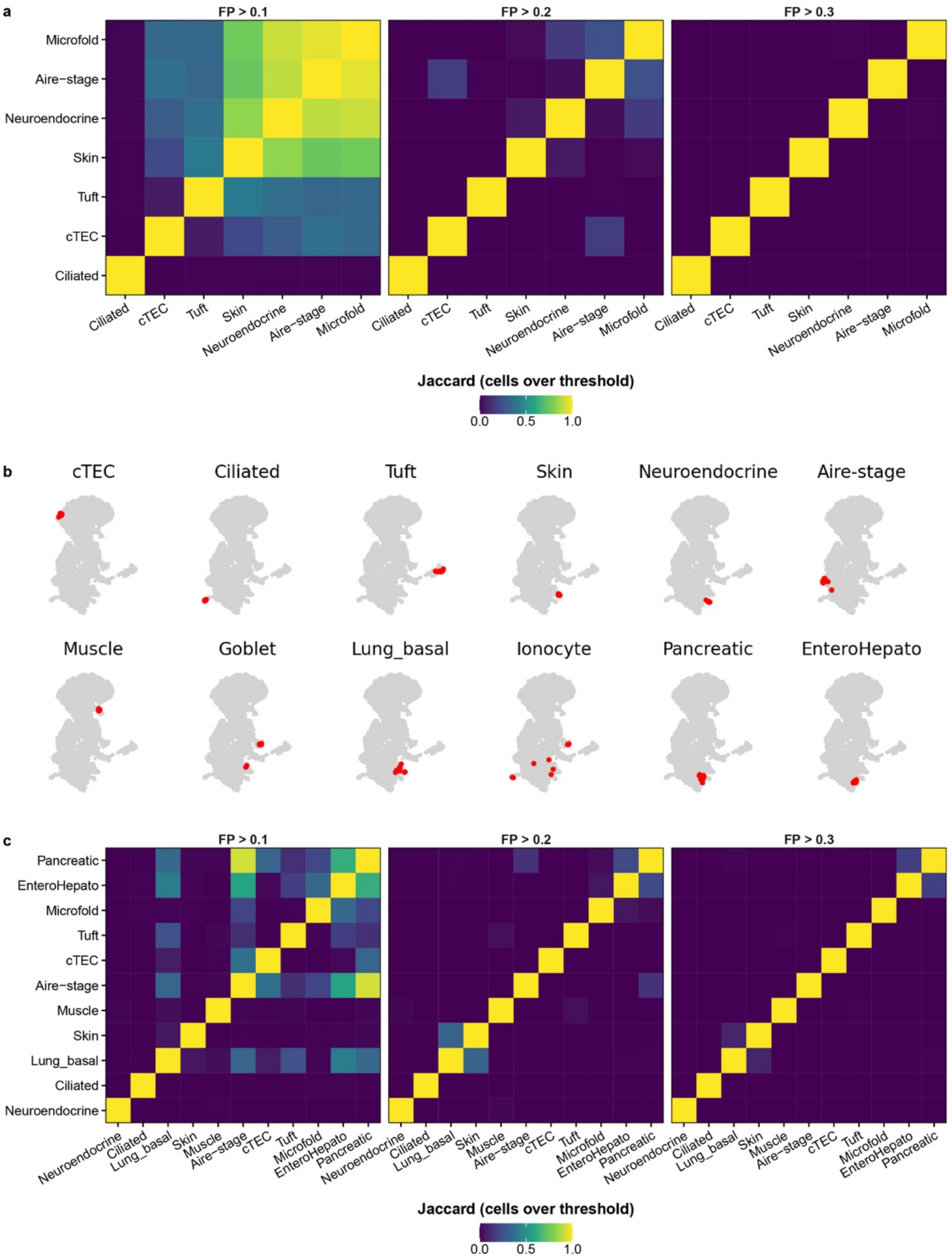

Supplementary Figure 3 | Fate trajectories of mouse TECs. a, Jaccard indices of sets of cells for pairs of fates. The set of cells for each fate comprises cells meeting the indicated fate probability thresholds for macrostates identified by CellRank (see Extended Data Figure 5). **b**, Location of cells selected as terminal cells via their respective signature scores (see Methods). These macrostates were considered for calculation of fate probabilities in **c**, with the exception of Goblet and Ionocyte because of their rarity and heterogeneity. **c**, Jaccard indices of sets of cells for pairs of fates. The set of cells for each fate comprises cells meeting the indicated fate probability thresholds for terminal states as defined in **b**.

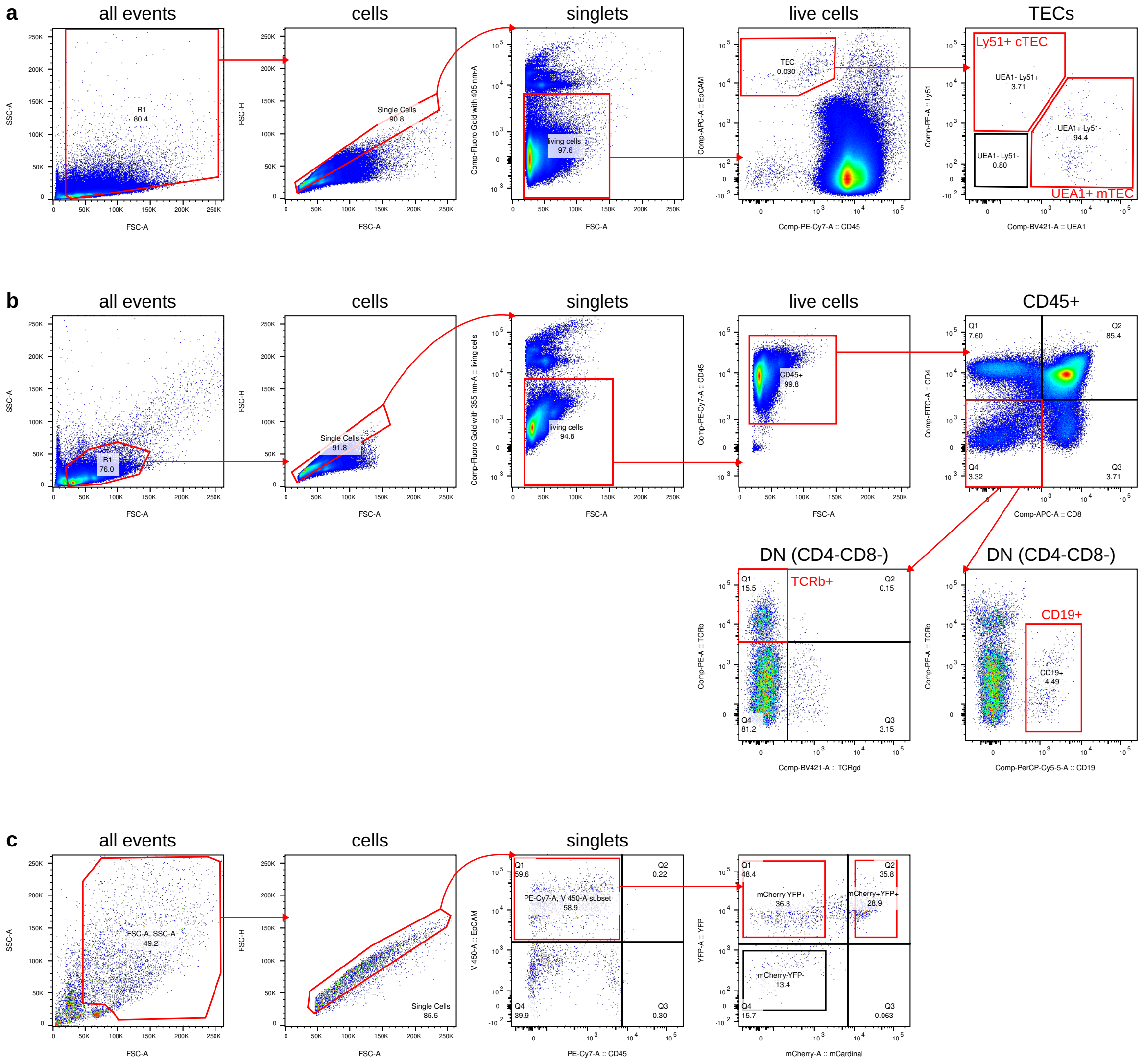

Supplementary Figure 4 | Gating strategy for thymus analysis. a, For analysis of thymic epithelial cells (TECs), thymic cell suspensions were gated for single cells, then gated for live cells using hydroxystilbamidine exclusion, and then the CD45^{-}Epcam^{+} fraction was resolved into Ly51^{-}UEA1^{+} mTECs, and Ly51^{+}UEA1^{-} cTECs. **b**, For analysis of thymocytes, live CD45^{+} cells were resolved into the four major thymocyte subsets using anti-CD4 and anti-CD8 antibodies. The gated CD4^{-}CD8^{-} double negative fraction was then resolved into TCRbeta^{+} immature thymocytes and CD19^{+} B cells. **c**, For preparative isolation of thymic epithelial cells (TECs), live CD45^{+} cell-depleted single thymic cells suspensions thymic cell suspensions were gated for CD45^{-}Epcam^{+} single cells, and then resolved into the two major indicated YFP^{+} fraction based on mCherry/mCradinal expression.

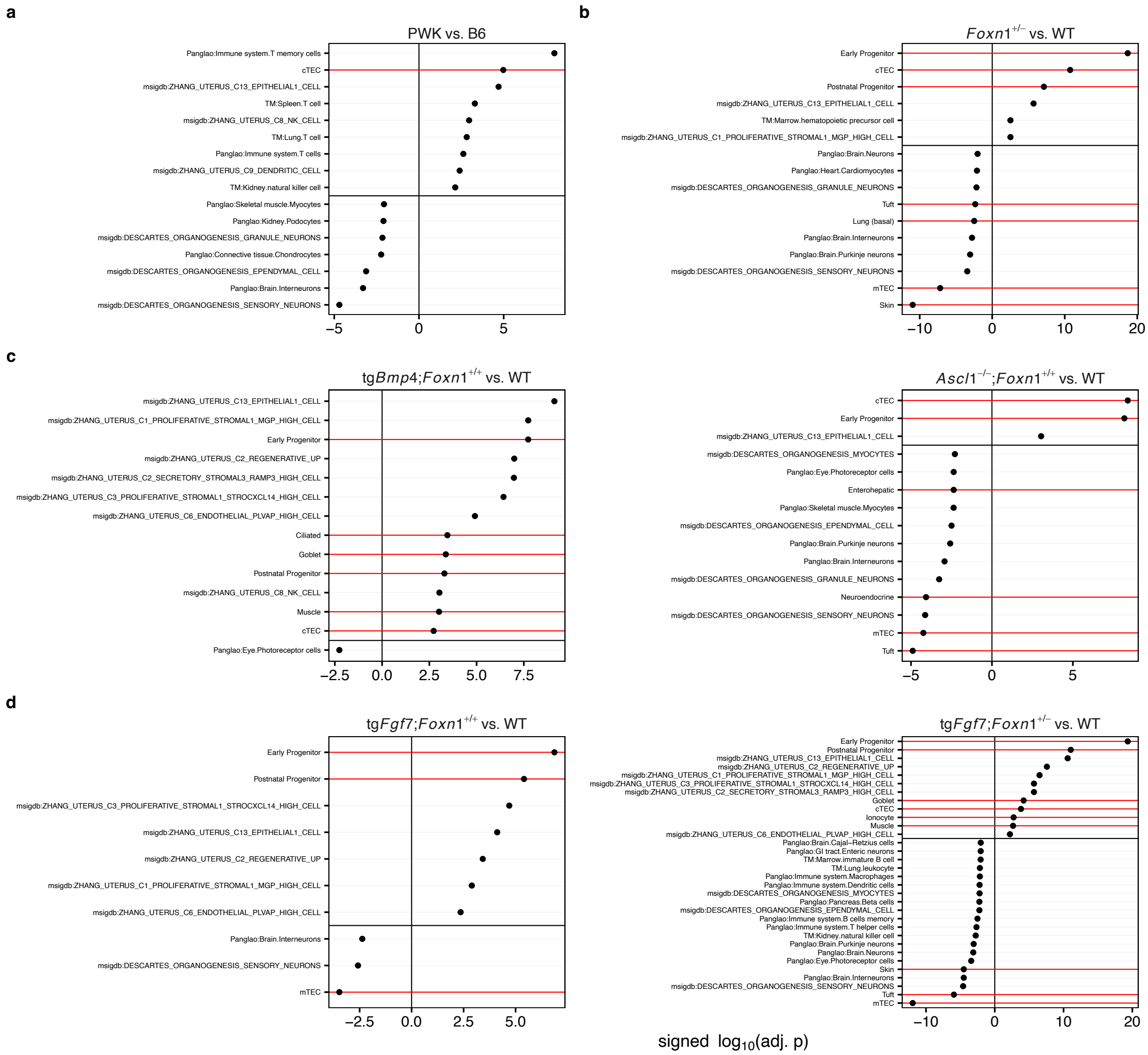

Supplementary Figure 5 | Top enriched signatures for **a**, TECs purified from PWK mice; **b**, from *Foxn1*$^{+/-}$ mice; **c**, from tg*Bmp4* and *Ascl1*$^{-/-}$ mice; **d**, tg*Fgf7*, and tg*Fgf7 Foxn1*$^{+/-}$ mice. See legend to Supplementary Figure 2 for details.

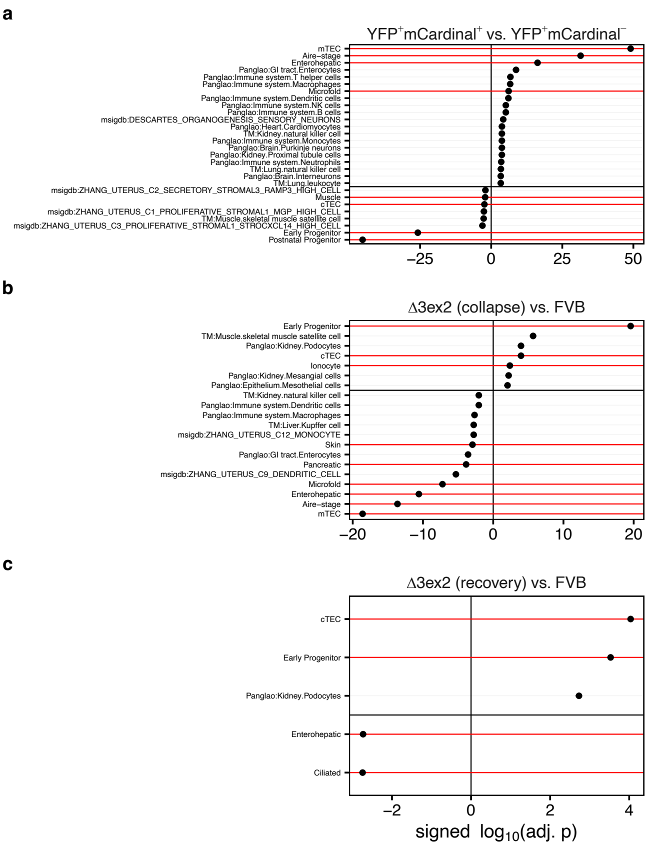

Supplementary Figure 6 | Top enriched signatures for **a**, TECs purified according to mCardinal signal, and **b**, **c**, TECs purified from Δ3ex2 mutants. See legend to Supplementary Figure 2 for details.

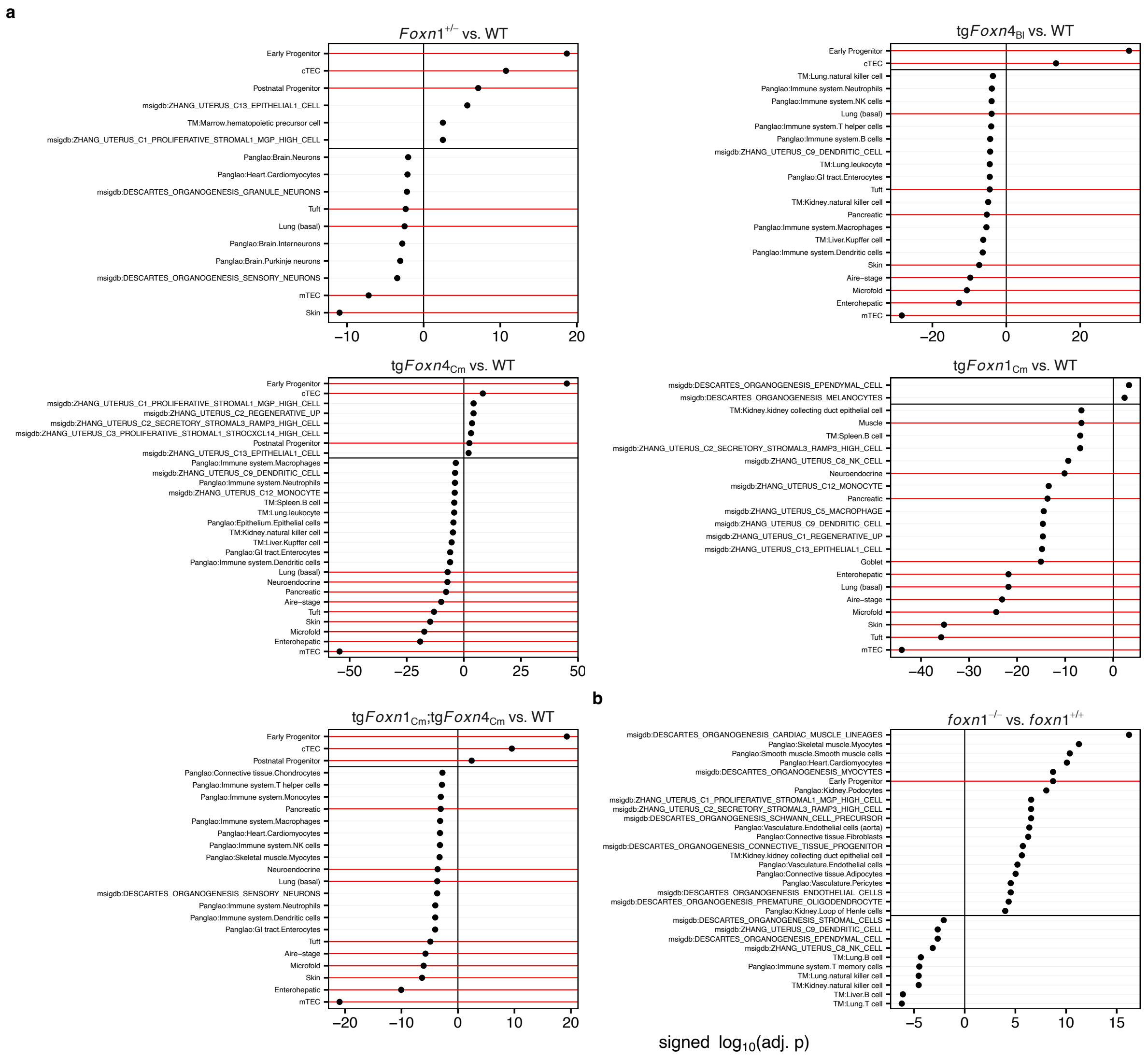

Supplementary Figure 7 | Analysis of evolutionarily relevant conditions. a, Top enriched signatures for TECs from *Foxn1*$^{-/-}$ or transgenic mice expressing ancient variants of *Foxn1* or *Foxn4*. **b**, Equivalent plot for TECs from *foxn1*$^{-/-}$ zebrafish. See legend to Supplementary Figure 2 for details.

a

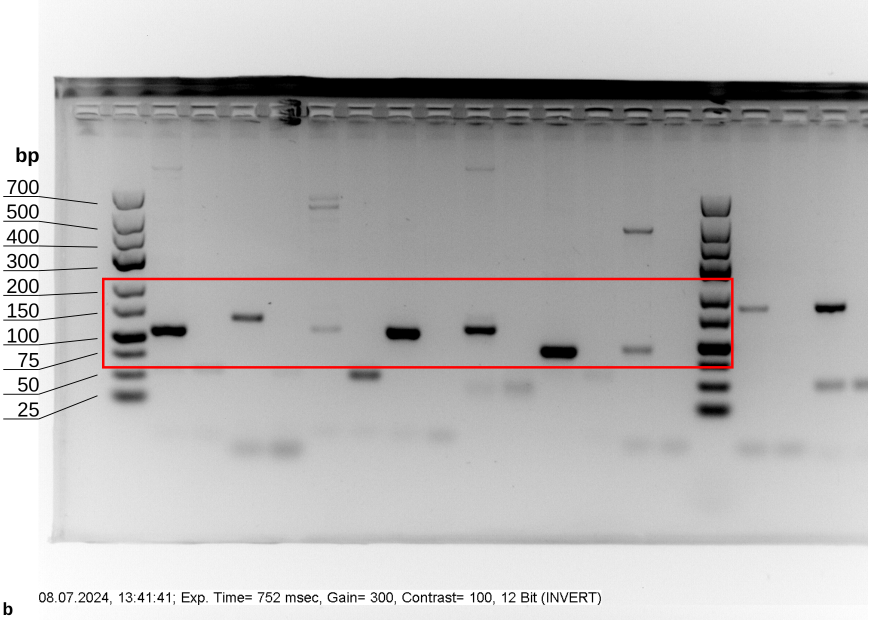
bp
700
500
400
300
200
150
100
75
50
25
08.07.2024, 13:41:41; Exp. Time= 752 msec, Gain= 300, Contrast= 100, 12 Bit (INVERT)

b

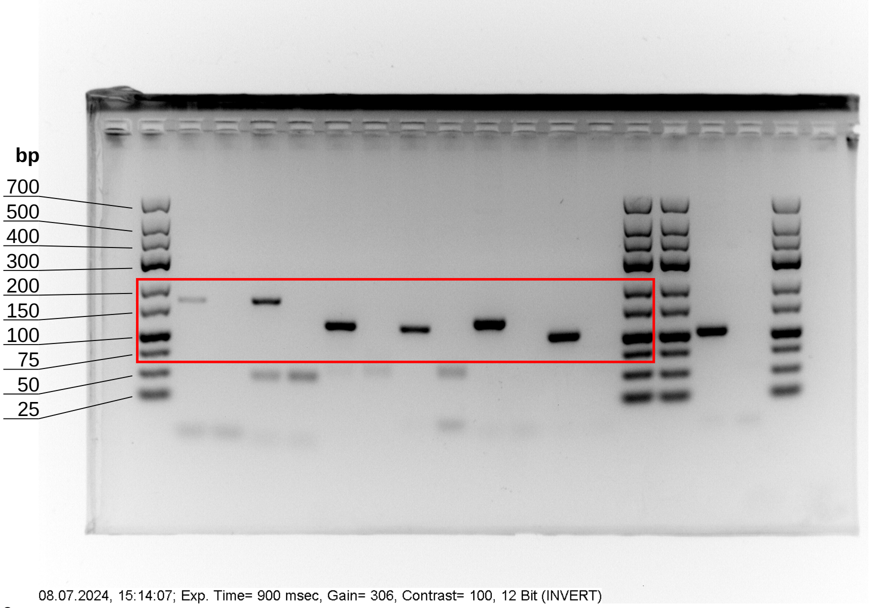
bp
700
500
400
300
200
150
100
75
50
25
08.07.2024, 15:14:07; Exp. Time= 900 msec, Gain= 306, Contrast= 100, 12 Bit (INVERT)

c

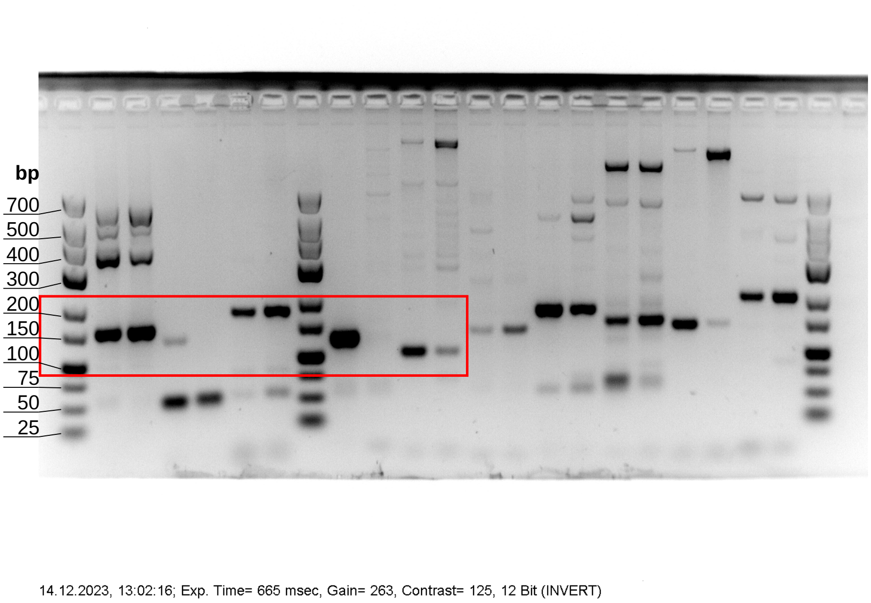
bp
700
500
400
300
200
150
100
75
50
25
14.12.2023, 13:02:16; Exp. Time= 665 msec, Gain= 263, Contrast= 125, 12 Bit (INVERT)

Supplementary Figure 8 | Uncropped gel electrophoresis assays. a, Refers to Extended Data Figure 10a, top panel. **b**, Refers to Extended Data Figure 10a, bottom panel. **c**, Refers to Extended Data Figure 11b, top panel. The cropped sections shown in the figures are indicated by red rectangles.
