# Supplementary material for: Developmental trajectory and evolutionary origin of thymic mimetic cells
Source: Nature. 2025 Jun 11;643(8073):1066–75. doi: 10.1038/s41586-025-09148-y (PMC12286861; doi:10.1038/s41586-025-09148-y)
Supplement: Supplementary file 2 — Reporting Summary [file 41586_2025_9148_MOESM2_ESM.pdf]

nature portfolio

Corresponding author(s): Thomas Boehm

Last updated by author(s): Apr 11, 2025

Reporting Summary

Nature Portfolio wishes to improve the reproducibility of the work that we publish. This form provides structure for consistency and transparency in reporting. For further information on Nature Portfolio policies, see our Editorial Policies and the Editorial Policy Checklist.

Statistics

For all statistical analyses, confirm that the following items are present in the figure legend, table legend, main text, or Methods section.

n/a	Confirmed	
☐	☒	The exact sample size (*n*) for each experimental group/condition, given as a discrete number and unit of measurement
☐	☒	A statement on whether measurements were taken from distinct samples or whether the same sample was measured repeatedly
☐	☒	The statistical test(s) used AND whether they are one- or two-sided<br>*Only common tests should be described solely by name; describe more complex techniques in the Methods section.*
☐	☒	A description of all covariates tested
☐	☒	A description of any assumptions or corrections, such as tests of normality and adjustment for multiple comparisons
☐	☒	A full description of the statistical parameters including central tendency (e.g. means) or other basic estimates (e.g. regression coefficient) AND variation (e.g. standard deviation) or associated estimates of uncertainty (e.g. confidence intervals)
☐	☒	For null hypothesis testing, the test statistic (e.g. *F*, *t*, *r*) with confidence intervals, effect sizes, degrees of freedom and *P* value noted<br>*Give P values as exact values whenever suitable.*
☒	☐	For Bayesian analysis, information on the choice of priors and Markov chain Monte Carlo settings
☒	☐	For hierarchical and complex designs, identification of the appropriate level for tests and full reporting of outcomes
☒	☐	Estimates of effect sizes (e.g. Cohen's *d*, Pearson's *r*), indicating how they were calculated

Our web collection on statistics for biologists contains articles on many of the points above.

Software and code

Policy information about availability of computer code

Data collection

FACS Diva Software v8.0.2, Summit 5.5 (MoFlow)

Data analysis

FlowJo 9.3.1 for flow cytometric analyses; GraphPad Prism 9.5.1

Raw data and the code to generate the figures for this manuscript can be found on Github (https://github.com/osthomas/mimetics_evodevo).

Analyses were run in conda (23.3.1) / mamba (1.4.2) environments with the following specifications:

R: r-base=4.2.3, r-here=1.0.1, r-tidyverse=1.3.2, r-ggplot2=3.5.1, r-remotes=2.4.2, r-devtools=2.4.5, r-matrix=1.6_1, r-matrixstats=1.0.0, r-patchwork=1.2.0, r-geomtextpath=0.1.4, r-dbplyr<=2.3.4, r-desctools=0.99.51, r-writexl=1.5.0, bioconductor-biomart=2.54.0, bioconductor-aucell=1.20.1, bioconductor-scran=1.26.0, bioconductor-scater=1.26.0, bioconductor-batchelor=1.14.0, bioconductor-complexheatmap=2.14.0

cellbender: python=3.7; pip: cellbender=0.3.0

sccoda: python=3.12.4; pip: pertpy=0.7.0, jax=0.4.30

scrnaseq: python=3.11.9, r-base=4.2.3, r-reticulate=1.38.0, quarto=1.5.57, r-tidyverse=1.3.2, r-ggplot2=3.5.1, r-remotes=2.4.2, r-devtools=2.4.5, r-patchwork=1.2.0, r-geomtextpath=0.1.4,

r-matrix=1.6_1, r-matrixstats=1.0.0, r-hdf5r=1.3.10, bioconductor-aucell=1.20.1, bioconductor-scran=1.26.0, bioconductor-scater=1.26.0, bioconductor-batchelor=1.14.0, bioconductor-scdblfinder=1.12.0, bioconductor-complexheatmap=2.14.0, bioconductor-zellkonverter=1.8.0, anndata=0.11.1, scanpy=1.10.3, cellrank=2.0.6; pip: igraph=0.11.8, leidenalg=0.10.2

zellkonverter: r-base=4.2.3, r-tidyverse=1.3.2, bioconductor-scran=1.26.0, bioconductor-scater=1.26.0, bioconductor-zellkonverter=1.8.0

For manuscripts utilizing custom algorithms or software that are central to the research but not yet described in published literature, software must be made available to editors and reviewers. We strongly encourage code deposition in a community repository (e.g. GitHub). See the Nature Portfolio guidelines for submitting code & software for further information.

Data

Policy information about availability of data

All manuscripts must include a data availability statement. This statement should provide the following information, where applicable:

- Accession codes, unique identifiers, or web links for publicly available datasets
- A description of any restrictions on data availability
- For clinical datasets or third party data, please ensure that the statement adheres to our policy

Primary read files and expression count files for the single cell RNA sequencing datasets were reported previously (Nusser A et al. Developmental dynamics of two bipotent thymic epithelial progenitor types. Nature 606, 165 (2022)) and are available from GEO (accession GSE106856). No restrictions apply.
Primary read files and expression count files for the bulk RNA sequencing datasets are available from GEO (accession numbers GSE272144; GSE272064; GSE272063). No restrictions apply.
Primary read files and expression count files for the single cell RNA sequencing datasets were reported previously (Nusser A et al. Developmental dynamics of two bipotent thymic epithelial progenitor types. Nature 606, 165 (2022)) and are available from GEO (accession GSE106856). No restrictions apply.
Primary read files and expression count files for the bulk RNA sequencing datasets are available from GEO (accession numbers GSE272144; GSE272064; GSE272063). No restrictions apply.
Primary read files and processed files for the single nuclear RNA sequencing datasets are available from GEO (accession GSE288957). No restrictions apply.
Background signature gene sets are available from PanglaoDB (https://panglaodb.se/markers.html), the Tabula Muris repository (https://github.com/czbiohub-sf/tabula-muris/tree/dedd8352d4348150e199162f966f7442976acdd3/22_markers) and MSigDB (https://www.gsea-msigdb.org/gsea/msigdb/mouse/genesets.jsp?collection=M8).

Research involving human participants, their data, or biological material

Policy information about studies with human participants or human data. See also policy information about sex, gender (identity/presentation), and sexual orientation and race, ethnicity and racism.

Reporting on sex and gender	n/a
Reporting on race, ethnicity, or other socially relevant groupings	n/a
Population characteristics	n/a
Recruitment	n/a
Ethics oversight	n/a

Note that full information on the approval of the study protocol must also be provided in the manuscript.

Field-specific reporting

Please select the one below that is the best fit for your research. If you are not sure, read the appropriate sections before making your selection.

☒ Life sciences ☐ Behavioural & social sciences ☐ Ecological, evolutionary & environmental sciences

For a reference copy of the document with all sections, see nature.com/documents/nr-reporting-summary-flat.pdf

Life sciences study design

All studies must disclose on these points even when the disclosure is negative.

Sample size	The sample sizes for each experiment are indicated in the figure legends. Sample sizes were based on our experience and accepted practice in the respective fields, balancing statistical robustness, resource availability and animal welfare. No statistical methods were used to predetermine sample size.
Data exclusions	No data were excluded
Replication	The sample sizes for each experiment are indicated in the figures or figure legends. RNAseq analysis was performed in a group-wise fashion, each group comprising at least two biological replicates; the group-wise comparison was not replicated. The results of several independent types of analyses are in agreement.
Randomization	Provided the transgenic status, and age of mice matched the experimental requirements, mice were randomly assigned to experimental groups.
Blinding	Blinding was not possible because the thymus phenotype, ie. the transgenic status of the respective mouse, is evident from flow cytometry, imaging analysis, or genotyping information.

Reporting for specific materials, systems and methods

We require information from authors about some types of materials, experimental systems and methods used in many studies. Here, indicate whether each material, system or method listed is relevant to your study. If you are not sure if a list item applies to your research, read the appropriate section before selecting a response.

Materials & experimental systems

n/a	Involved in the study
☐	☒ Antibodies
☒	☐ Eukaryotic cell lines
☒	☐ Palaeontology and archaeology
☐	☒ Animals and other organisms
☒	☐ Clinical data
☒	☐ Dual use research of concern
☒	☐ Plants

Methods

n/a	Involved in the study
☒	☐ ChIP-seq
☐	☒ Flow cytometry
☒	☐ MRI-based neuroimaging

Antibodies

Antibodies used

Flow Cytometry:
Anti-EpCAM, host: Rat IgG2a, κ , conjugation: APC, clone: G8.8, supplier: BioLegend, cat#: 118214, dilution: 1:1000
Anti-EpCAM, host: Rat IgG2a, κ, conjugation: Biotin, clone: G8.8, supplier: BioLegend, cat#: 118204, dilution: 1:1000
Anti-CD45, host: Rat IgG2b, κ, conjugation: PE/Cy7, clone: 30-F11, supplier: BioLegend, cat#: 103114, dilution: 1:2000
Anti-Ly51 (alias: BP-1), host: Rat / IgG2a, kappa, conjugation: PE, clone: 6C3, supplier: ThermoFisher/eBioscience, cat#: 12-5891-82, dilution: 1:1600
UEA1, host: N/A, conjugation: FITC, clone: -, supplier: VectorLabs, cat#: FL-1061 , dilution: 1:1000
UEA1, host: N/A, conjugation: Biotin, clone: -, supplier: VectorLabs, cat#: B-1065-2 , dilution: 1:600
Anti-CD4, host: Rat IgG2b, κ , conjugation: FITC, clone: GK1.5, supplier: BioLegend, cat#: 100406, dilution: 1:1000
Anti-CD8a, host: Rat / IgG2a, kappa , conjugation: APC, clone: 53-6.7, supplier: ThermoFisher/eBioscience, cat#: 17-0081-82, dilution: 1:800
Anti-CD19, host: Rat / IgG2a, kappa, conjugation: PerCP/Cy5.5, clone: eBio1D3, supplier: ThermoFisher/eBioscience, cat#: 45-0193-82, dilution: 1:500
Anti-CD19, host: Rat / IgG2a, kappa, conjugation: PE/Cy7, clone: eBio1D3, supplier: ThermoFisher/eBioscience, cat#: 25-0193-82, dilution: 1:1000
Anti-CD45R (B220), host: Rat / IgG2a, kappa, conjugation: Biotin, clone: RA3-6B2, supplier: ThermoFisher/eBioscience, cat#: 13-0452-82, dilution: 1:200
Anti-IgM, host: Rat / IgG2a, kappa, conjugation: PE, clone: II/41, supplier: ThermoFisher/eBioscience, cat#: 12-5790-82, dilution: 1:300
Anti-CD93, host: Rat / IgG2b, kappa, conjugation: APC, clone: AA4.1, supplier: ThermoFisher/eBioscience, cat#: 17-5892-81, dilution: 1:300
anti-TCRb, host: Armenian hamster / IgG, conjugation: PE, clone: H57-597, supplier: ThermoFisher/eBioscience, cat#: 12-5961-82, dilution: 1:400
streptavidin, host: N/A, conjugation: FITC, clone: -, supplier: ThermoFisher/eBioscience, cat#: 11-4317-87, dilution: 1:1000

streptavidin, host: N/A, conjugation: eFluor450, clone: -, supplier: ThermoFisher/eBioscience, cat#: 48-4317-82, dilution: 1:1000
Anti-CD45, host: rat IgG2b, conjugation: MicroBeads, clone: 30-F11.1, supplier: Miltenyi Biotec, cat#: 130-052-301, dilution: 1:20, max. 1x10e8 cells/ml
Anti-Ter-119, host: rat IgG2b, conjugation: MicroBeads, clone: -, supplier: Miltenyi Biotec, cat#: 130-049-901, dilution: 1:20, max. 1x10e8 cells/ml

ISH:
Anti-Digoxigenin, host: sheep IgG, conjugation: alkaline phosphatase (AP), clone: polyclonal (Fab fragments), supplier: Roche, cat#: 11093274910, dilution: 1:2000
Anti-Digoxigenin, host: sheep IgG, conjugation: horseradish peroxidase (POD), clone: polyclonal (Fab fragments), supplier: Roche, cat#: 11207733910, dilution: 1:300
Anti-Fluorescein, host: sheep IgG, conjugation: horseradish peroxidase (POD), clone: , supplier: Roche, cat#: 11426346910, dilution: 1:300

Validation

All antibodies used in this study were sourced from commercial suppliers. Details about their validation strategies are available from:

BioLegend: https://www.biolegend.com/en-us/quality/product-development
ThermoFisher:
https://www.thermofisher.com/de/en/home/life-science/antibodies/invitrogen-antibody-validation.html
VectorLabs: https://vectorlabs.com/browse/antibodies/
Miltenyi Biotec: https://www.miltenyibiotec.com/US-en/products/macs-antibodies/antibody-validation.html

All antibodies were suitable for the applications as used in this study according to the manufacturers.

Anti-EpCAM (APC):
FC - Quality tested
Product Information: https://www.biolegend.com/ja-jp/products/apc-anti-mouse-cd326-ep-cam-antibody-4974

Anti-EpCAM (Biotin):
FC - Quality tested
Product Information:
https://www.biolegend.com/fr-ch/products/biotin-anti-mouse-cd326-ep-cam-antibody-4725

Anti-CD45:
FC - Quality tested
Product Information: https://www.biolegend.com/en-us/products/pe-cyanine7-anti-mouse-cd45-antibody-1903

Anti-Ly51 (alias: BP-1):
The 6C3 antibody has been tested by flow cytometric analysis of mouse bone marrow cells.
Product Information:
https://www.thermofisher.com/antibody/product/CD249-BP-1-Antibody-clone-6C3-Monoclonal/12-5891-82

UEA1:
Applications: Immunofluorescence, Glycobiology
Product Information: https://vectorlabs.com/products/fluorescein-ulex-europaeus-agglutinin/

UEA1 (Biotin):
Applications: Immunohistochemistry / Immunocytochemistry, Immunofluorescence, Blotting Applications, Elispot, ELISAs, Glycobiology
Product Information: https://vectorlabs.com/products/biotinylated-ulex-europaeus-agglutinin/

Anti-CD4:
FC - Quality tested
Product Information: https://www.biolegend.com/fr-ch/products/fitc-anti-mouse-cd4-antibody-248

Anti-CD8a:
Applications Tested: The 53-6.7 antibody has been tested by flow cytometric analysis of mouse thymocyte or splenocyte suspensions.
Product Information:
https://www.thermofisher.com/antibody/product/CD8a-Antibody-clone-53-6-7-Monoclonal/17-0081-82

Anti-CD19 (PerCP/Cy5.5):
Applications Tested: This eBio1D3 (1D3) antibody has been tested by flow cytometric analysis of mouse splenocytes.
Product Information:
https://www.thermofisher.com/antibody/product/CD19-Antibody-clone-eBio1D3-1D3-Monoclonal/45-0193-82

Anti-CD19 (PE/Cy7):
Applications Tested: This eBio1D3 (1D3) antibody has been tested by flow cytometric analysis of mouse splenocytes.
Product Information:
https://www.thermofisher.com/antibody/product/CD19-Antibody-clone-eBio1D3-1D3-Monoclonal/25-0193-82

Anti-CD45R:
Applications Tested: The RA3-6B2 antibody has been tested by flow cytometric analysis of mouse splenocytes.
Product Information:
https://www.thermofisher.com/antibody/product/CD45R-B220-Antibody-clone-RA3-6B2-Monoclonal/13-0452-82

Anti-IgM:
Applications Tested: This II/41 antibody has been tested by flow cytometric analysis of mouse bone marrow cells.
Product Information:
https://www.thermofisher.com/antibody/product/IgM-Antibody-clone-II-41-Monoclonal/12-5790-82

Anti-CD93:
Applications Tested: The AA4.1 antibody has been tested by flow cytometric analysis of mouse bone marrow and splenocyte cells.
Product Information:
https://www.thermofisher.com/antibody/product/CD93-AA4-1-Antibody-clone-AA4-1-Monoclonal/17-5892-81

anti-TCRb:
Applications Tested: The H57-597 antibody has been tested by flow cytometric analysis of mouse thymocytes and splenocytes.
Product Information:
https://www.thermofisher.com/antibody/product/TCR-beta-Antibody-clone-H57-597-Monoclonal/12-5961-82

streptavidin (FITC):
Reported Application: Flow Cytometric Analysis, Immunocytochemistry, Immunohistochemical Staining of Frozen Tissue Sections
Product Information:
https://www.thermofisher.com/order/catalog/product/11-4317-87?SID=srch-srp-11-4317-87

streptavidin (eFluor450):
Reported Application: Flow Cytometric Analysis
Product Information:
https://www.thermofisher.com/order/catalog/product/48-4317-82?SID=srch-srp-48-4317-82

Anti-CD45:
Mouse CD45 MicroBeads were developed for the positive selection or depletion of leukocytes from lymphoid and non-lymphoid tissues.
Product Information:
https://www.miltenyibiotec.com/DE-en/products/cd45-microbeads-mouse.html#130-052-301

Anti-Ter-119:
Anti-Ter-119 MicroBeads are suitable for positive selection or depletion of mouse erythrocytes or erythroid progenitors from lymphoid tissues.
Product Information:
https://www.miltenyibiotec.com/DE-en/products/anti-ter-119-microbeads-mouse.html#130-049-901

Anti-Digoxigenin:
Suitable for histochemistry according to product documentation
Product Information:
https://www.sigmaaldrich.com/FR/fr/product/roche/11093274910?srsltid=AfmBOop9KqUAP9_sx1EBxC88b93gHrQdb34DUlVaQut61-u7VivAvfpv

Anti-Digoxigenin:
Suitable for histochemistry according to product documentation
Product Information:
https://www.sigmaaldrich.com/FR/fr/product/roche/11207733910?srsltid=AfmBOood4LuUNBU3J7AkCs1QOOe1d56p61vcvZ7kpssbSI_P-MEdWXNa

Anti-Fluorescein:
Suitable for histochemistry according to product documentation
Product Information:
https://www.sigmaaldrich.com/FR/fr/product/roche/11426346910?srsltid=AfmBOoqgCTmub8Nik3Uci19GbkM4OWdKNWyr9aiBVgKJtncmjm7CDBo5

Animals and other research organisms

Policy information about studies involving animals; ARRIVE guidelines recommended for reporting animal research, and Sex and Gender in Research

Laboratory animals

C57BL/6 mice were maintained in the Max Planck Institute of Immunobiology and Epigenetics. Foxn1-/-, Foxn1:Cre, Rosa26-LSL-EYFP, Foxn1:mCardinal, Ascl1fl/fl, Foxn1:Bl_Foxn4, Foxn1:Cm_Foxn4, and

Foxn1:Cm_Foxn1 as well as Foxn1:Fgf7 transgenic mice have been described previously. The Foxn1:Bmp4 transgene was created by Thomas Schlake and B.K. by inserting a cDNA fragment corresponding to nucleotides 497–1729 in GenBank accession number NM_007554.3 as a NotI fragment into pAHB1434. The Δ3ex2 Foxn1 deletion mutant (internal designation Chi6) transgene was generated by deletion of nucleotides 504-728 of mouse Foxn1 cDNA (Genbank accession number NM_008238.2) and insertion as a NotI fragment into pAHB1434. To generate transgenic mice, constructs were linearized and injected into FVB pronuclei according to standard protocols. The Foxn1Δ3ex2 mice were bred to a Foxn1-deficient background. Genotyping information is summarized in Supplementary Table 2. Mice carrying the original nu mutation (CByJ.Cg-Foxn1nu/J) were purchased from Charles River and used for snRNAsq experiments.
The zebrafish line carrying an internal deletion of the foxn1 gene was described.

Mice were analysed at the age of 4-6 weeks, unless otherwise stated.

Adult zebrafish (3 months of age) were used for experiments.

Wild animals: No wild animals were used.

Reporting on sex: Phenotypes did not vary according to sex; for zebrafish embryos, sex determination is not possible

Field-collected samples: Ammocoete larvae of Lampetra planeri were caught from the field in the Freiburg region (Riedgraben, March-Neuershausen) (no precise age can be determined; body length 8-10 cm); juvenile Scyliorhiunus canicula specimens (directly after hatching) were kindly supplied by Markéta Kauka (Max Planck Institute for Evolutionary Biology, Plön, Germany); jjuvenile bamboo sharks (Chiloscyllium punctatum) were purchased from a local pet shop. Upon arrival at the laboratory, lampreys and sharks were euthanized using 0.02% tricaine methanesulfonate.

Ethics oversight: All animal experiments were performed in accordance with the relevant guidelines and regulations, approved by the review committee of the Max Planck Institute of Immunobiology and Epigenetics and the Regierungspräsidium Freiburg, Germany (mice: licenses 35-9185.81/G-12/85; 35-9185.81/G-16/67; zebrafish: license 35–9185.81/G-14/41). Ammocoete larvae of Lampetra planeri were caught from the wild in the Freiburg region (Riedgraben, March-Neuershausen) under permission by the local governmental authority (LandratsamtBreisgau-Hochschwarzwald, license 420.1.13-2024-034414).

Note that full information on the approval of the study protocol must also be provided in the manuscript.

Plants

Seed stocks: *Report on the source of all seed stocks or other plant material used. If applicable, state the seed stock centre and catalogue number. If plant specimens were collected from the field, describe the collection location, date and sampling procedures.*

Novel plant genotypes: *Describe the methods by which all novel plant genotypes were produced. This includes those generated by transgenic approaches, gene editing, chemical/radiation-based mutagenesis and hybridization. For transgenic lines, describe the transformation method, the number of independent lines analyzed and the generation upon which experiments were performed. For gene-edited lines, describe the editor used, the endogenous sequence targeted for editing, the targeting guide RNA sequence (if applicable) and how the editor was applied.*

Authentication: *Describe any authentication procedures for each seed stock used or novel genotype generated. Describe any experiments used to assess the effect of a mutation and, where applicable, how potential secondary effects (e.g. second site T-DNA insertions, mosiacism, off-target gene editing) were examined.*

Flow Cytometry

Plots

Confirm that:

☒ The axis labels state the marker and fluorochrome used (e.g. CD4-FITC).

☒ The axis scales are clearly visible. Include numbers along axes only for bottom left plot of group (a 'group' is an analysis of identical markers).

☒ All plots are contour plots with outliers or pseudocolor plots.

☒ A numerical value for number of cells or percentage (with statistics) is provided.

Methodology

Sample preparation: Thymic epithelial cells have the surface phenotype EpCAM+/CD45−; thus, cell surface staining was performed using anti-EpCAM (G8.8), conjugated with APC (1:1000, BioLegend) or anti-EpCAM (G8.8), conjugated with biotin (1:1000, BioLegend), in combination with streptavidin, conjugated with eFluor 450 (1:1000, eBioscience), and anti-CD45 (30-F11), conjugated with PE Cy7 (1:2000, BioLegend) at 4°C in PBS supplemented with 0.5% BSA and 0.02% NaN3. In order to differentiate cells with past and acute Foxn1 expression, triple-transgenic Foxn1:Cre; RosaR26SLSYFP; Foxn1:mCardinal mice were used for cell sorting. Cells with past expression of Foxn1 were sorted as EpCAM+YFP+ mCardinal- cells, whereas cells with acute Foxn1 expression were sorted as EpCAM+YFP+ mCardinal+ cells. CD45−EpCAM+ cells [after negative enrichment using anti-CD45 magnetic-activated cell sorting (MACS) beads and

anti–Ter-119 MACS beads, Miltenyi Biotec] were sorted directly into TRI reagent (T9424, Sigma-Aldrich). Cell sorting was carried out using the MoFlow instrument (Dako Cytomation-Beckman Coulter) controlled with the Summit (5.5) software. Analytical flow cytometry was performed for TECs as follows: anti-EpCAM (G8.8), conjugated with APC (1:1000, BioLegend); anti-Ly51 (alias BP-1; 6C3), conjugated with PE (1:1600, eBioscience); UEA1, conjugated with FITC (1:1000, Vector Labs) or UEA1, conjugated with biotin (1:600, Vector Labs), in combination with streptavidin, conjugated with eFluor 450 (1:1000, eBioscience). When analysis of haematopoietic fractions was desired, thymocyte suspensions were prepared in parallel by mechanical liberation, best achieved by gently pressing thymic lobes through 40 µm sieves. Cell surface staining [anti-CD45 (30-F11), conjugated with PE/Cy7 (1:2000, BioLegend); anti-CD4 (GK1.5), conjugated with FITC (1:1000, BioLegend); anti-CD8a (53-6.7), conjugated with APC (1:800, eBioscience); anti-TCRβ (H57-597), conjugated with PE (1:400, eBioscience); anti-CD19 (eBio1D3), conjugated with PerCP/Cy5.5 (1:500, eBioscience) or PE/Cy7 (1:1000, eBioscience); anti-B220 (alias CD45R; RA3-6B2), conjugated with biotin (1:200, eBioscience); anti-IgM (II/4.1), conjugated with PE (1:300, eBioscience), anti-CD93 (alias C1qRp; AA4.1), conjugated with APC (1:300, eBioscience); streptavidin conjugated with eFluor 450 or FITC (1:1000, eBioscience)] was performed at 4°C in PBS supplemented with 0.5% BSA and 0.02% NaN3. Flow cytometry experiments were evaluated using FACSDiva (8.0.2) and FlowJo (9.3.1) software. The relevant gating strategies are shown in Supplementary Figure 4.

Instrument

BD Fortessa II; MoFlow; both from Dako Cytomation-Beckman Coulter

Software

FACS Diva Software v8.0.2, Summit 5.5 (MoFlow), FlowJo 9.3.1 for flow cytometric analyses
Data analysis was carried out in conda environments

Cell population abundance

Purity was determined by running a purity check of the sorted populations after the sort was completed.

Gating strategy

All samples were initially gated using forward and side scatter to identify events corresponding to cells, doublets are excluded by gating on single cells using forward scatter height vs. area, alive cells were selected by negativity for the viability dye Fluoro Gold, the follow gating steps are according to the marker genes described in the manuscript

☒ Tick this box to confirm that a figure exemplifying the gating strategy is provided in the Supplementary Information.
